# The first identified skeletal collection of the Azores archipelago, Portugal

**DOI:** 10.1093/fsr/owaf046

**Published:** 2025-11-14

**Authors:** Félix Rodrigues, António Félix Rodrigues, Vítor Matos, Armando Mendes, Eugénia Cunha, Maria Teresa Ferreira

**Affiliations:** Laboratory of Forensic Anthropology, Centre for Functional Ecology, Department of Life Sciences, University of Coimbra, Coimbra, Portugal; Research Centre for Anthropology and Health, Department of Life Sciences, University of Coimbra, Coimbra, Portugal; Investigation Institute for Agrarian Technologies and Environment, University of the Azores, Angra do Heroísmo, Portugal; Research Centre for Anthropology and Health, Department of Life Sciences, University of Coimbra, Coimbra, Portugal; Laboratory of Biological Anthropology, Department of Biology, School of Science and Technology, University of Évora, Évora Portugal; Centre for Humanistic Studies, University of the Azores, Ponta Delgada, Portugal; Laboratory of Forensic Anthropology, Centre for Functional Ecology, Department of Life Sciences, University of Coimbra, Coimbra, Portugal; South Branch, National Institute of Legal Medicine and Forensic Sciences (INMLCF), Lisboa, Portugal; Laboratory of Forensic Anthropology, Centre for Functional Ecology, Department of Life Sciences, University of Coimbra, Coimbra, Portugal

**Keywords:** forensic sciences, forensic anthropology, human remains, Atlantic islands

## Abstract

Identified skeletal collections play a crucial role in anthropology, as the biographic information they contain enables the development and adaptation of population-specific identification methods, namely for biological profile (sex, age-at-death, stature, and ancestry). In Portugal, aside from academic systematic research, these collections also permit the training of new professionals while also providing an ethical destination for unclaimed skeletal remains. As of now, mainland Portugal has nine identified skeletal collections representing populations of a great part of its territory. Nevertheless, the population from the Portuguese insular territories, with its unique migration history and distinctive cultural and environmental contexts, has remained unrepresented until now. This paper presents the first identified skeletal collection from the Azores archipelago (CEI/Açores) that results from a pioneering effort between the two Terceira island municipalities (Angra do Heroísmo and Praia da Vitória) and academic researchers in anthropology. A protocol, established in March of 2023 and still ongoing, enabled the integration of unclaimed human skeletal remains from municipal cemeteries in the CEI/Açores. This collection was assembled not only for scientific research purposes but also with the aim of preventing their cremation or deposition in mass graves, which could lead to the loss of identity and hinder future recovery. Currently, the CEI/Açores is housed and legally possessed by Praia da Vitória municipality and includes 75 identified skeletons of individuals who were born on Terceira island, where they also died between 1978 and 2013. The majority of them (87%) are almost complete or relatively complete. Females (56%, *n* = 40) are slightly more represented than males (44%, *n* = 35). The overall age-at-death ranges between 26 and 97 years (mean = 71.47 ± 15.09). Specifically, the ages range for males is 26–89 years (mean = 65.42 ± 16.45), and for females, it is 39–97 years (mean = 76.83 ± 11.55). Various bone lesions, spanning traumatic, neoplastic, degenerative, and potentially infectious origins, were observed, alongside the presence of prosthetics, pacemakers, and other medical devices. Although the CEI/Açores is still in its early stages and is currently undergoing expansion, it already presents significant research potential, not only at the local level but also on a global scale.

## Introduction

Human skeletal collections, whether identified or not, play an essential role in forensic and biological anthropology. The biological information they provide can be used to infer life conditions and death circumstances of an individual or a population. For instance, identified skeletal collections (composed of individuals with known biographic information, typically including biological sex and age-at-death) have enabled the development of forensic identification methods, primarily for biological profile estimation. Morphological and metric differences in the skeleton, observed through systematic studies conducted in identified collections worldwide, have yielded a vast array of methods for estimating age-at-death, biological sex, population affinity, and stature [1–3]. Nevertheless, population differences require adaptation in these methods, and therefore, the assembly of new worldwide collections capable of reflecting population variability is of utmost importance [4, 5]. As of now, Portugal has nine identified skeletal collections [4–9].

Portuguese law stipulates that, after a minimum of 3 years following burial, if entirely skeletonized, remains can be exhumed and graves can be reused [10]. The specific duration between burials in the same grave may be increased depending on the cemetery’s capacity, as these management practices and criteria are delegated to the municipalities. The fate of unclaimed remains is also determined by the most suitable management solution for the municipalities [11]. Whenever a grave is designated for reuse, municipalities contact the closest surviving family members of the deceased to enquire about their preferences for the fate of the exhumed remains. In the absence of instructions, such as purchasing the grave or transferring the bones to a family grave or a private ossuary, the remains are considered legally abandoned [10]. In such cases, they may be placed in common graves, general ossuaries, subjected to cremation [11], or donated to universities for teaching and research purposes [12]. It is important to note that, with the exception of the last, these practices imply the loss of identity and preclude the possibility of future recovery [13–15].

Assembling identified skeletal collections with unclaimed or abandoned remains has been a more ethical approach as it helps maintain the identity associated with the skeleton while also allowing for potential future inhumation upon request, supporting scientific research, and contributing to the training of forensic anthropology following ethical international guidelines [16–18].

While Portuguese identified collections cover much of the continental territory, making Portugal one of the leading countries in the world in this area [5], the Madeira archipelago lacks representation, and the Azores archipelago has been unrepresented until the present. In this paper, we present and provide an overview of the first identified skeletal collection from the Azores (CEI/Açores). This collection is currently in the process of development and expansion, and we highlight its regional and global importance.

### Reasoning behind the identified skeletal collection of the Azores (CEI/Açores)

The first identified skeletal collection from the Azores, herein CEI/Açores, serves several of the previously mentioned purposes, particularly ethical and scientific, while also possessing its own unique characteristics.

Firstly, it comprises individuals who lived and died in the Azores, a group of islands where the first Portuguese settlers (>500 years ago) were part of a founder effect [19]. Nonetheless, the initial difficulty in further populating this region led the Portuguese crown to grant land and privileges to other groups as well, for instance Jews, slaves from Cape Verde, Guinea, and São Tomé as well as English, French, Spanish, and Flemish. Previous genetic studies have been conducted on the Azorean population that support the historical sources as they revealed genetic proximity to mainland Portugal [19–21]. Furthermore, a study involving the diversity of 15 STR markers had shown similar genetic diversity values between islands but some differences to mainland Portugal, which could be simultaneously attributed to the genetic bottleneck, possible mutations through time and genetic contributions of other population groups such as the ones mentioned previously [22]. Demography studies also revealed high consanguinity coefficients in the Azores [23] that translate into particularly high frequencies of neurodegenerative diseases such as Machado Joseph [24]. Despite the identification of these historical, demographic, and genetic specificities, their impact on skeletal changes, often reflected in secular trends, remains largely unexplored. So far, anthropological studies have been constrained to a limited number of archaeological excavations, primarily focused on religious buildings, scattered across various islands [25, 26], mostly dating from the initial centuries of the Azores settlement [26]. The human remains yielded from these excavations are also scattered through five of the nine islands, which makes comprehensive studies difficult [25]. Furthermore, insularly identified skeletal collections are exceedingly rare on a global scale [5]. Only one, namely in Crete (Greece), contains individuals that died in a recent past [27].

Secondly, and as previously stated, the anthropological framework in the Azores is very limited, especially in the field of forensic anthropology where no studies have been conducted yet. Establishing an identified skeletal collection will open up new research opportunities in multiple fields of anthropology, both on a regional and global scale.

## Materials and Methods

### The first steps to develop the collection

In 2023, a protocol was legally established between the two municipalities of Terceira Island (Angra do Heroísmo and Praia da Vitória) with the recognition of the need to provide a respectful and dignified outcome for abandoned remains, while also contributing to the scientific advancement of the region. The official protocol was established between the two municipalities, with Praia da Vitória municipality as the legal possessor of the collection and the first author, an academic researcher in anthropology from the Department of Life Sciences, University of Coimbra, serving as its curator. Besides legal possession by the municipality, the management, research strategies, and decisions regarding third-party access requests are delegated to the curator. The protocol also takes several other factors into consideration, including maintaining the anonymity of the donated individuals to third parties unless legally requested, the option of making them accessible to municipalities or returning them to the cemetery upon a family’s request, the exclusion of massive destructive bone analysis, and ensuring the respectful handling and storage of the remains.

Basic biographical information for each skeleton was collected through the cemetery records, which include sex, age-at-death, place-of-birth, date-of-death and burial, grave slot, and municipality of last residence. In few cases, occupation, cause of death, and life events such as physical violence or accidents that resulted in bone lesions are also known. The latter were voluntarily provided by individuals who knew the deceased and were present during the exhumation procedure. Nevertheless, this information should be considered carefully as it is narratives. Additionally, the exhumations of unclaimed skeletons were accompanied by the curator in order to gather supplementary information about the burial conditions, such as grave and urn types, burial depth, inhumation position, personal belongings, and other scientifically relevant details. This presence also ensures the correct recovery of all the remains, allows the collection of grave soil samples for future analysis and includes cemetery workers as an active part of the scientific production.

**Figure 1:**
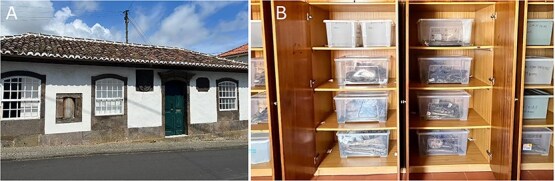
(A) Façade view of Casa da Roda (Praia da Vitória), where the CEI/Açores is housed. (B) Cleaned and dried skeletons are stored in individual labelled boxes.

After exhumation, each skeleton was stored in an individualized box containing its biographical information and transported to the laboratory, located at Casa da Roda, in Praia da Vitória, a historical building owned by the municipality that displays the suitable conditions for anthropological investigation (Figure 1A). Upon arrival, an acronym was attributed to each skeleton based on the order of exhumation. For instance, the first exhumed skeleton was classified as “CEIAç_esq1”. In addition, comprehensive physical and digital records, including detailed description of the custody chain, were created. Skeletons were classified according to completeness as follows: very incomplete (when >50% of the skeleton is absent), relatively complete (when 25%–50% of the skeleton is absent), almost complete (<25% of the skeleton is absent), or complete (when all bones are present). The cleaning process is gradually taking place, using a toothbrush and distilled water since future non-destructive chemical analysis is planned. After cleaned and dried, bones were placed on shelves by numerical order (Figure 1B).

## Results

### State of completeness, sex, and age-at-death distribution

At present, the collection comprises 75 skeletons. According to the methodology mentioned above, 39 (52%) are almost complete, 26 (35%) relatively complete, and 10 (13%) very incomplete. A detailed assessment of skeletal preservation and bone quality is still pending since some of the individuals are yet to be cleaned, as this process is time-consuming. Females are slightly more represented (53%, *n* = 40) than males (47%, *n* = 35). All individuals were born and died in the Azores, with their deaths spanning from 1978 to 2013. Nevertheless, 73% (*n* = 55) died between 2010 and 2013. Table 1 displays the distribution of individuals in the collection by sex and age-at-death groups.

**Table 1:** Distribution of age-at-death and sex among individuals in the CEI/Açores.

Sex	Age-at-death groups (years)						Total
	20–29	30–49	50–69	70–89	≥90	Unknown	
Male	1	5	11	13	–	5	35
Female	–	2	5	26	3	4	40
Total	1	7	16	39	3	9	75

As observable in Table 1, all individuals were adults, predominantly elderly. However, 24 of them died before reaching 70 years old. Overall, the ages-at-death range from 26 to 97 years, with a group mean age-at-death of 71.47 ± 15.09 years. Males’ ages-at-death ranged from 26 to 89 years, with a mean of 65.42 ± 16.45 years. For females, the ages-at-death varied between 39 and 97 years, with a mean of 76.83 ± 11.55 years. When compared with the most recent collections in Portugal, where age-at-death distribution is known, namely the identified collections in Coimbra (CEI/XXI) and Évora, the Azorean sample shows lower mean values for age-at-death than Coimbra for both sexes [7], and lower than Évora for males. As for the Évora collection, it includes non-adult individuals of both sexes, which lowers these values [4]. In the case of the nine individuals with unknown age-at-death, the absence of this information can be attributed to the cemetery records in Praia da Vitória, which only include references to the sex and year of death. Gathering age-at-death information for these individuals is planned for the near future, but it will require research in municipal archives. The absence of non-adults in this collection can be explained by the Portuguese low mortality rates in this age group [28], and the fact that most cemeteries on Terceira island have dedicated sections with ample space for non-adult burials, eliminating the need for grave exhumation for reuse. Nevertheless, these ages reflect only death patterns in the population and contrast inversely with the living populations. In fact, the living Azorean population is younger than the one in mainland Portugal, with only 16.5% >65 years old *vs.* 23.4%, respectively [29].

### Places of birth, last residence, and cemetery of burial

Out of the total sample, 66 skeletons originated from the Conceição Cemetery, the municipal cemetery of Angra do Heroísmo, while nine came from the municipal cemetery of Praia da Vitória. The Conceição Cemetery serves five “freguesias”, which are small administrative localities within the municipality that do not have their own cemetery, namely Nossa Senhora da Conceição, São Bento, Santa Luzia, Sé, and São Pedro. The Praia da Vitória cemetery serves only one “freguesia”, namely Santa Cruz of Praia da Vitória. The majority of the individuals were buried in the cemetery that served the area of their last residence (65%, *n* = 49) or in the cemetery that served their place of birth (12%, *n* = 9). Three individuals (4%), all from the same family (a mother and two sons), were exhumed from a private grave at Conceição municipal cemetery that was considered legally unclaimed after 15 years. Municipal cemetery regulations permit the burial of individuals in these three conditions (in the cemetery serving the individual’s place of birth, in the cemetery serving their last residence area, or in a private family grave, even if it is not located in either of those areas) [11]. However, Portuguese law predicts other conditions that are yet to be included in municipal regulations but in fact can occur. For instance, a social funeral. This could be the case of 14 individuals (19%) that were interred in a municipal cemetery without fulfilling the three criteria mentioned above. The rationale behind their burial in a specific cemetery remains unclear and only inferences can be drawn based on last residence and place of birth records and material findings discovered during the exhumation process. These include hospital blankets involving the remains, autopsy signs, and other medical devices normally utilized during hospitalization. Smaller Azorean islands, where six of these individuals had their last residence, lack hospitals. For instance, São Jorge island. In emergency cases, patients can be transferred and treated on Terceira island. In case of death where family instructions are absent, a social funeral will take place in the cemetery that serves the area of the hospital, as it is the case of Conceição cemetery. Nevertheless, further investigation is needed in order to confirm or reject these hypotheses.

### Bone lesions, chirurgical interventions, medical devices, and causes of death

A detailed paleopathological analysis is still dependent on the cleaning process, nevertheless, despite this ongoing process, a considerable number of pathologies, surgical interventions, and medical devices have already been identified within the collection. These findings include two cases of autopsy, both involving relatively young adult males (26 and 42 years old at the time of death), and one case involving both antemortem and perimortem trauma in a young adult male (36 years old at death). Additionally, other noteworthy cases include another case of antemortem trauma to the skull and a potential femoral antemortem traumatic lesion, three hip prostheses, two cases of skull osteolytic lesions, one case with an osteoblastic lesion in the tibia and another on the right ilium, three cases of left sacroiliac fusion, six cases of femur and tibia periosteal new bone formation, seven cases of dental prostheses crafted from various materials, two pacemakers, and two cases of Kirschner wires in the sternum, and one in the right ulna.

**Figure 2:**
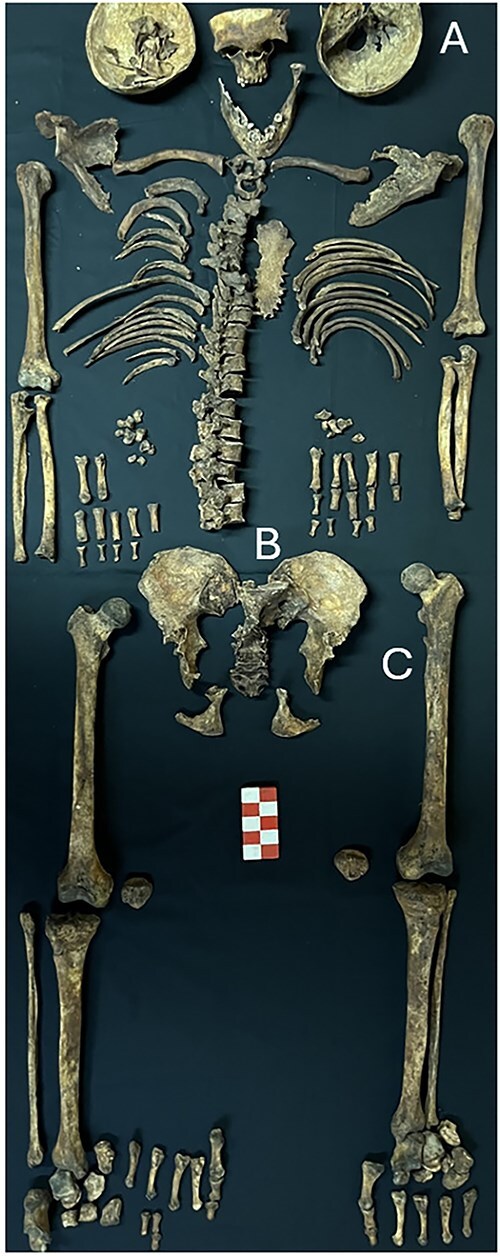
Skeleton CEIAç_esq1 exhibiting (A) autopsy signs, (B) left sacroiliac fusion, and (C) a possible antemortem traumatic lesion to the left femur.

Furthermore, older individuals within the collection display diverse signs of degenerative changes, including osteoarthritis. Notably, five cases exhibit lesions consistent with diffuse idiopathic skeletal hyperostosis. Figure 2 illustrates a 42-year-old male (CEIAç_esq1) exhibiting autopsy signs and pathological conditions on the left innominate and femur.

While these observations provide valuable insights into the health and medical history of the individuals in the collection, a comprehensive pathological analysis and a future attempt to collect individual medical history records promise to unveil even more details.

Regarding causes of death, they are only known for four individuals as they were freely mentioned by individuals who knew the deceased and attended the exhumations. They are an accidental traumatic event (CEIAç_esq2), a suicide through herbicide ingestion (CEIAç_esq15), a pancreatic cancer (CEIAç_esq24), and a breast cancer (CEIAç_esq40). This information is particularly important since causes of death are absent in cemetery records.

## Discussion and Conclusion

The establishment of the Identified Skeletal Collection in the Azores (CEI/Açores) represents a pioneering effort to enrich our understanding of the distinctive biological, cultural, and historical intricacies intrinsic to island populations while also permitting the adaptation and development of population specific forensic identification methods. This endeavour holds the potential to illuminate broader patterns of human adaptation and health from a global vantage point and contribute to the application of adapted forensic methods, and therefore more precision, in present-day forensic cases in the Azores. The growth of this new collection is essential for facilitating comprehensive statistical and individualized analyses, with the current assembly of 75 individuals expected to expand to 200 within the next 2 years. This anticipated growth is particularly crucial in small island settings, where even unclaimed skeletal remains can intertwine with detailed personal histories, elevating the depth and individuality of our analyses. Meticulous curation, storage, and documentation are vital for preserving the enduring value of this collection.

In the contemporary era marked by heightened human mobility and global interconnectedness, the once-pronounced relevance of isolated evolutionary mechanisms and genetic adaptations in small island environments has undergone significant transformation. However, this evolving context does not diminish the scholarly intrigue surrounding island populations which often grapple with unique health challenges and exhibit distinct patterns influenced by factors such as limited healthcare resources [30], idiosyncratic dietary practices, and specific environmental exposures [31].

The examination of skeletal remains from modern island populations presents a compelling opportunity to reveal the prevalence of specific diseases, nutritional deficiencies, and other health-related phenomena while also offering an ethical destination for unclaimed remains, facilitating cemeteries space management, and potentiating local scientific research. Through the investigation of these populations, we deepen our comprehension of the broader biological terrain encompassing public health, epidemiology, and human biology. Additionally, we delve into the intricate interplay between genetics, environment, health outcomes, and skeletal morphological variation. The insights derived from the study of modern island populations hold promise not solely for the immediate island milieu but also for analogous regions grappling with similar biological and health challenges. These insights collectively contribute to a shared global understanding of human health and adaptation in our interconnected world.
